# Supplementary material for: Polyploid cancer cells reveal signatures of chemotherapy resistance
Source: Oncogene. 2024 Nov 22;44(7):439–49. doi: 10.1038/s41388-024-03212-z (PMC11810791; doi:10.1038/s41388-024-03212-z)
Supplement: Supplementary file 1 — Supplementary Methods and Figures [file 41388_2024_3212_MOESM1_ESM.pdf]

Supplementary Methods

Single cell picking: Eppendorf TransferMan NK2 micromanipulator was used to collect the cell of interest in a 20 µM micropipette (for control cells) or a 100 µM micropipette (for treated surviving cells). The cell was transferred to a PCR tube containing 0.2% TritonX-100 and RNAse Inhibitor. Single cells were stored in -80°C for downstream DNA or RNA sequencing.

Immunofluroescent staining: Slides were removed from the -80°C and immediately fixed with 2% paraformaldehyde for 20 minutes. Slides were then blocked with 2% BSA and then incubated overnight at 4°C with a primary antibody cocktail consisting of mouse IgG1/Ig2a anti-human cytokeratins (CK) 1, 4, 5, 6, 8, 10, 13, 18, and 19 (clones: C-11, PCK-26, CY-90, KS-1A3, M20, A53-B/A2, C2562, Sigma, St. Louis, MO, USA), mouse IgG1 anti-human CK 19 (clone: RCK108, GA61561-2, Dako, Carpinteria, CA, USA), and mouse EpCAM.

Viability: VivaFix cell viability dye (Bio-Rad catalog 1351115) was utilized to evaluate cell permeability. Cells were treated with chemotherapy and allowed to recover. Following recovery cells were lifted with 1x versene (ThermoFisher catalog 15040066), spun down, and resuspended in 1x PBS with VivaFix dye. Cells were then plated on Marienfeld glass slides, incubated at 37°C for 30 minutes, briefly washed in PBS, and then fixed with 2% paraformaldehyde for 20 minutes. Cells were then stained with DAPI, EPI cocktail, and CD45, and imaged via high content scanning.

Single cell copy number profiling: Briefly, single cell whole genome amplification was performed via the WGA4 kit (Sigma) and NEB Ultra FS II was used for library preparation with 50 ng of starting material. Cells were sequenced at a depth of 1-2 million reads on an Illumina HiSeq 4000. Sequencing reads were aligned with BWA-MEM to the hg38 reference. Count data was segmented via the R package DNACopy (version 1.70.0), and median segmented ratio values were reported.

Supplementary Tables

Table S1: Bone marrow patient PFS data and treatment history.

Table S2: Copy number concordance values for patient data.

Table S3: Copy number concordance values for cell line data.

Table S4: Convergent polyploid gene set with the 309 genes that are in common between MDA-MB-231 and PC3 polyploid cancer cells.

Supplementary Figures: S1-S13

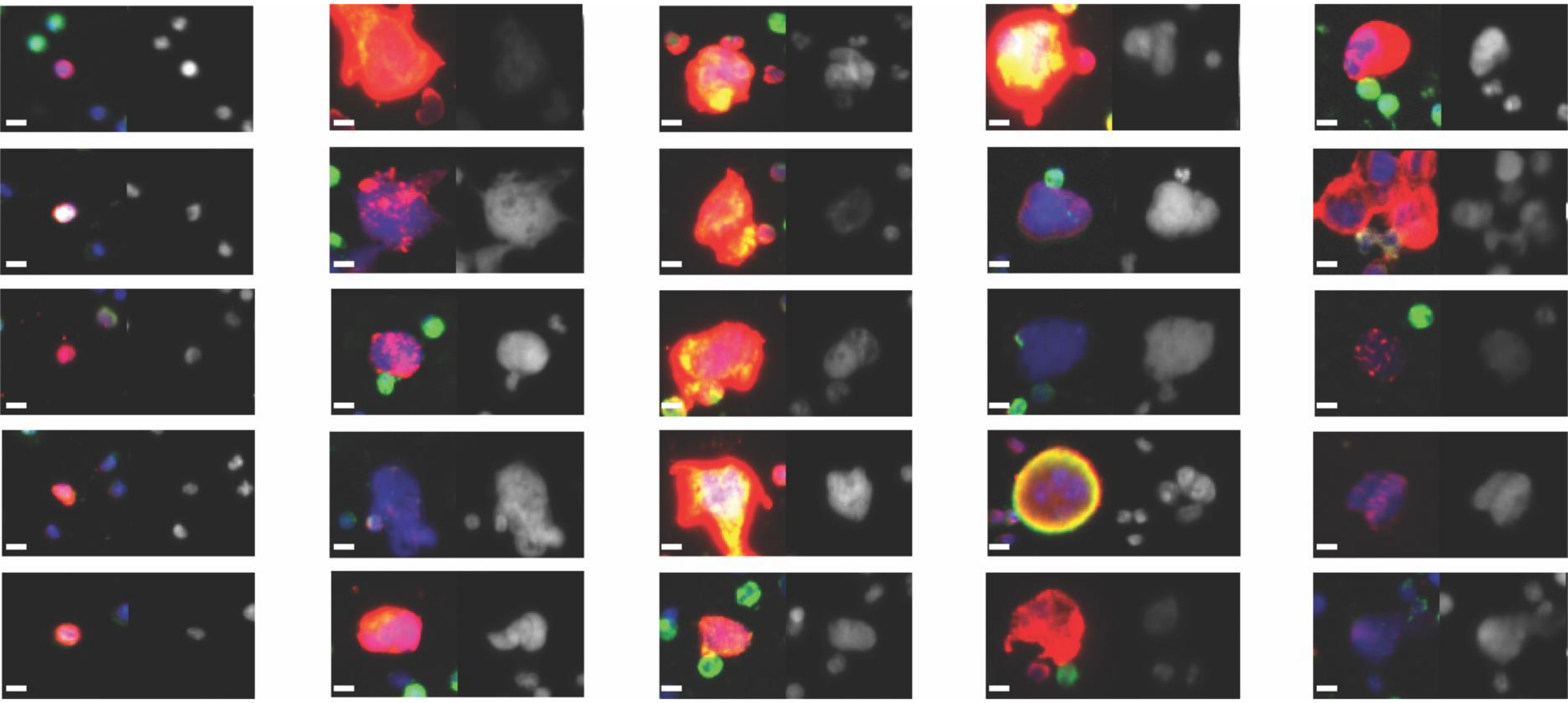

Figure S1: Representative gallery of merged composite images (left) and DAPI images (right) of typical CTCs (first column) and CTC-IGC (columns 2-5) found in bone marrow aspirate of late-stage prostate cancer patients. Scale bars are set to 15 µM. Epithelial channel (pan-CK + EpCAM) is in red, DAPI is in blue, CD45 is in green, and vimentin is in white in the merged composite image.

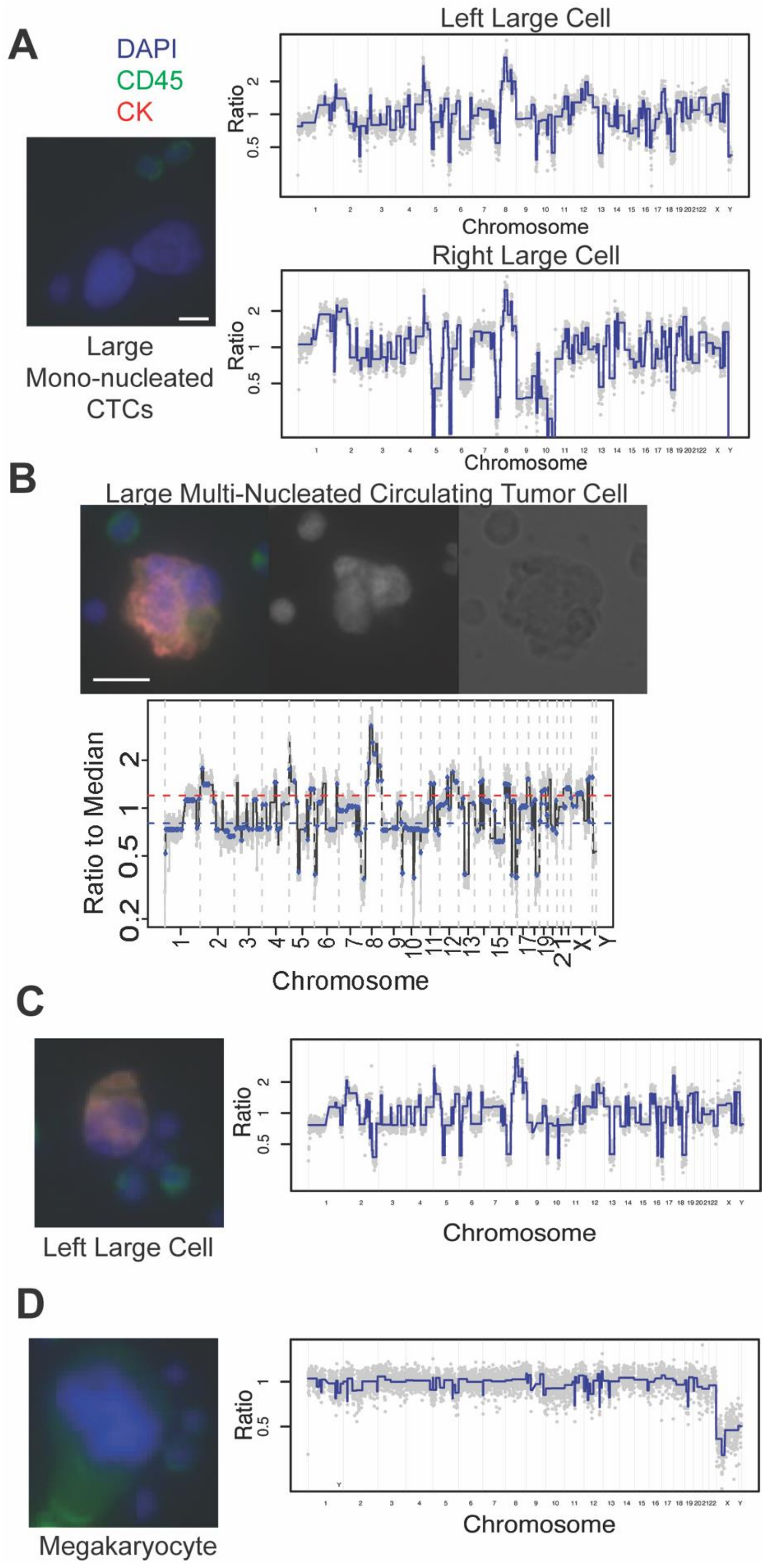

Figure S2: (A-C) Representative copy number profiles of CTC-IGC and (D) a non-altered megakaryocyte found in the bone marrow of a prostate cancer patient. The Megakaryocyte shows a noisy profile with a ratio around 1, while CTC-IGCs display distinct breakpoints.

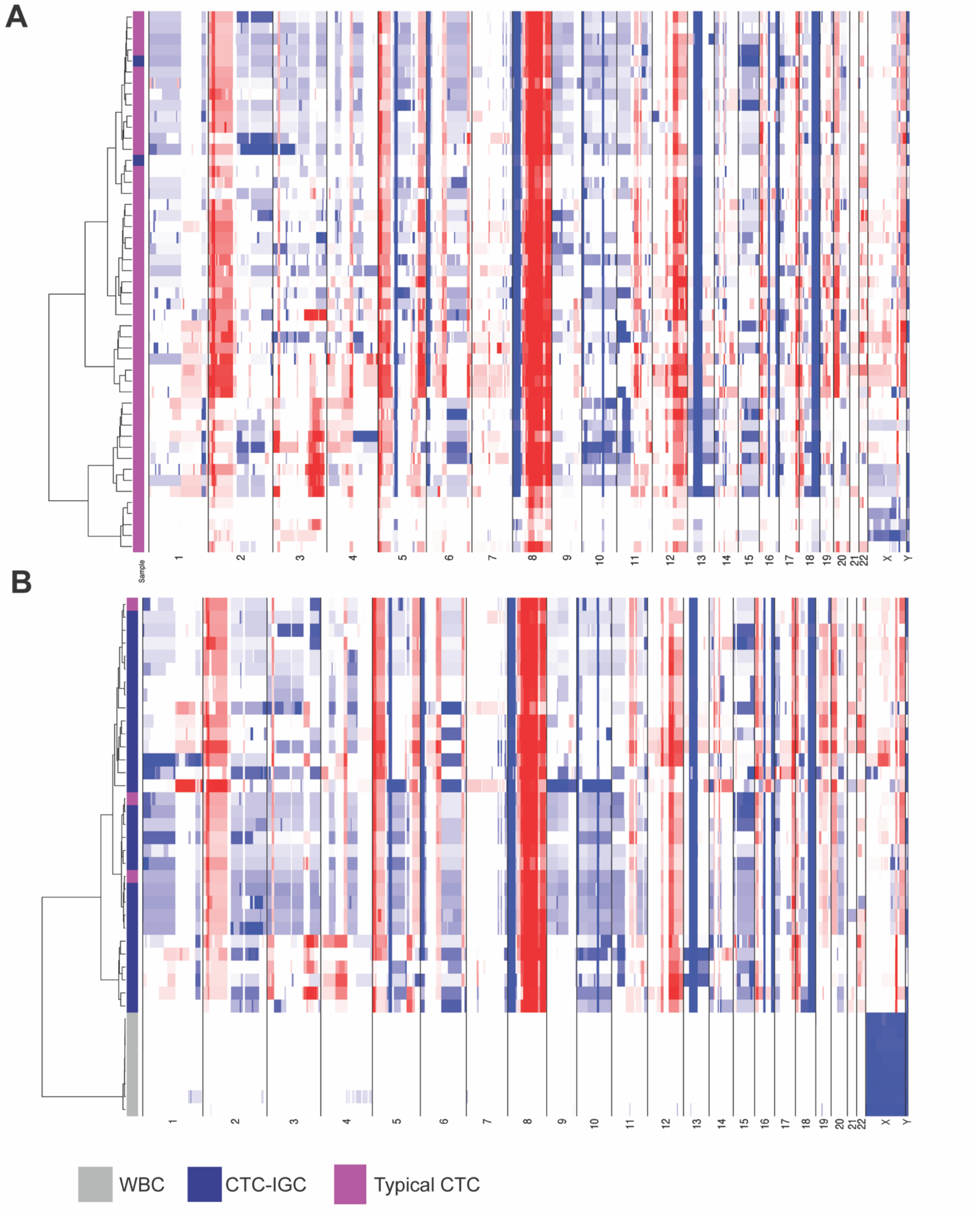

Figure S3: Copy number profiles from patient in figure 1 showing clonality of CTCs and CTCs-IGC. White blood cells are megakaryocytes. (A) is single end sequenced data (1x50bp) and (B) is paired end data (2x150bp)

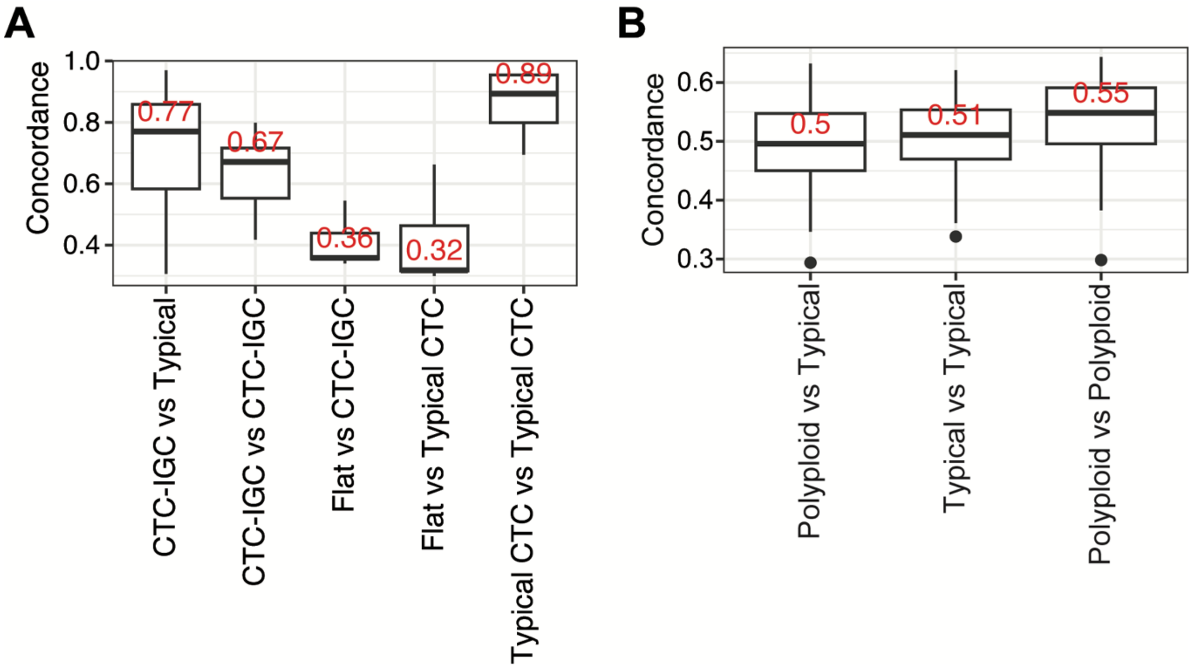

Figure S4: Copy number concordances between each condition for patient cells in (A) and cell lines in (B). In (A) CTC-IGC vs Typical shows that the median concordance between a single CTC-IGC and a single typical CTC is 77%, while comparing CTC-IGCs to each other (single CTC-IGC vs. single CTC-IGC) has a median concordance of 67%. Wilcoxon testing showed no significant differences in comparing CTC-IGC vs typical CTCs. Heterogeneity is observed in cell lines in (B), with a median concordance around 50%.

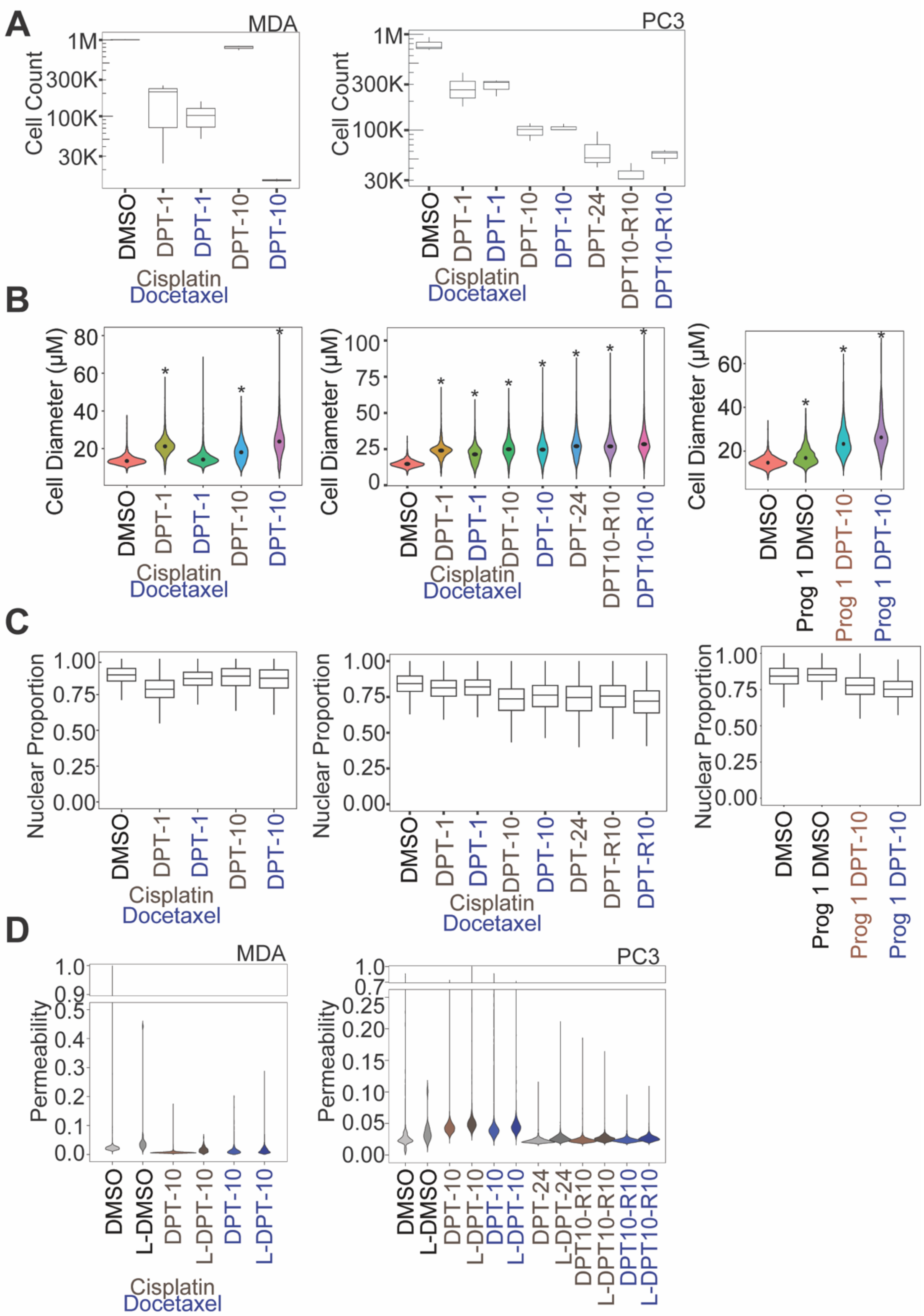

Figure S5: Image analysis of polyploid PC3 and MDA-MB-231 cancer cells. (A) Cell count for DMSO control and recovery conditions for MDA-MB-231 (left) and PC3 (right) cells. (B) Cell diameter calculations for MDA-MB-231 (left), PC3 (middle), and PC3 progeny-1 (right) cells. (C) Nuclear proportion (nuclear diameter / cellular diameter) for MDA-MB-231 (left), PC3 (middle), and PC3 progeny-1 (right) cells. (D) Permeability intensity for MDA-MB-231 (left) and PC3 (right) cells. "L" corresponds to largest 15% cells and shows no difference in permeability.

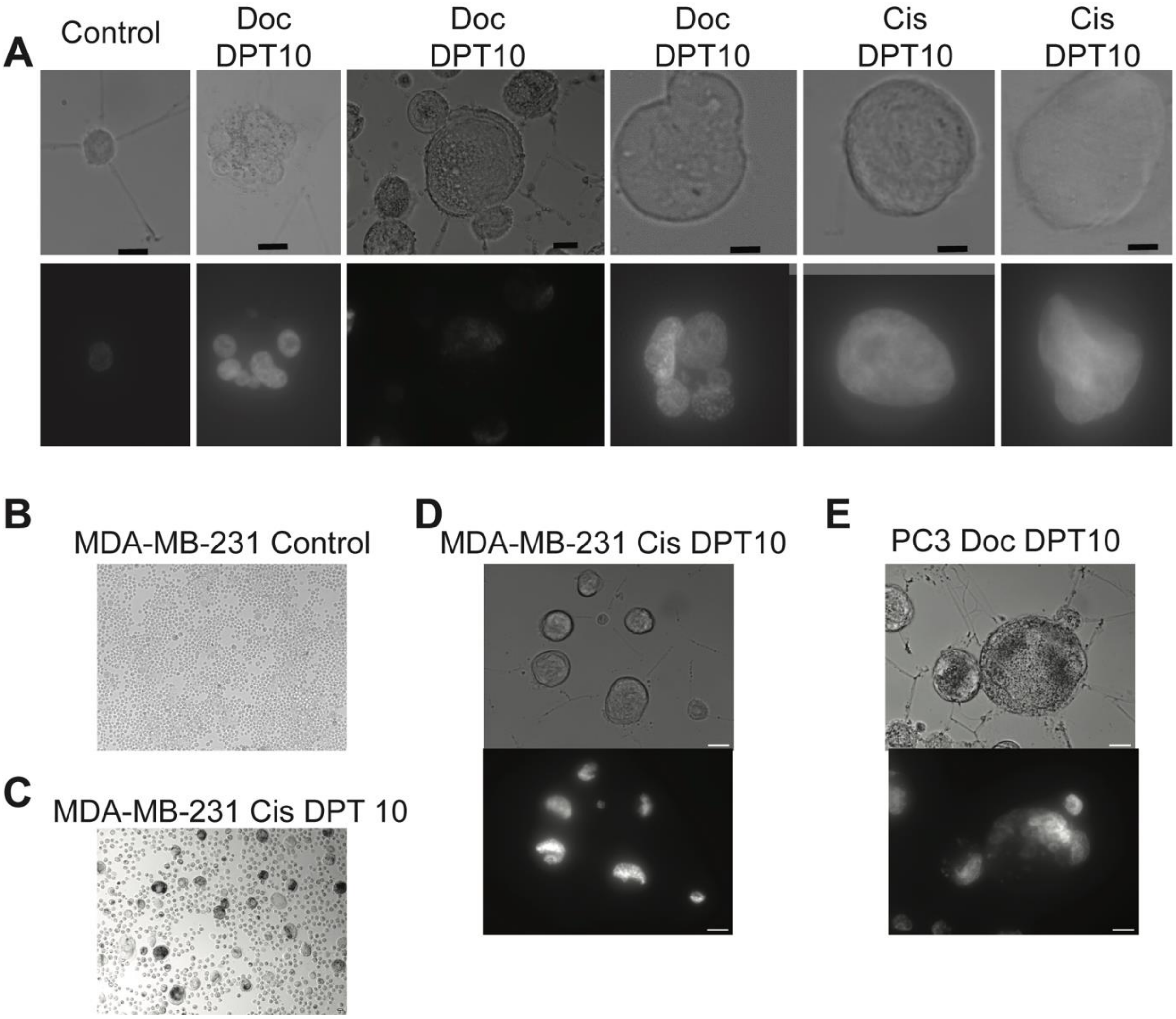

Figure S6: (A) Representative 40x cell line images for control (DMSO) and polyploid cell lines. Top row represents bright field and bottom row depicts the DAPI channel. Scale bars set to 23 µM. (B) 10x image of MDA-MB-231 control cells. Scale bars set to 20 µM. (C) 10x image of cisplatin 10 day recovered MDA-MB-231 cell lines. Scale bars set to 20 µM. (D) 40x image of cisplatin 10 day recovered MDA-MB-231 cell lines. Scale bars set to 20 µM. (E) 40x image of docetaxel 10 day recovered PC3 cell lines. Scale bars set to 20 µM.

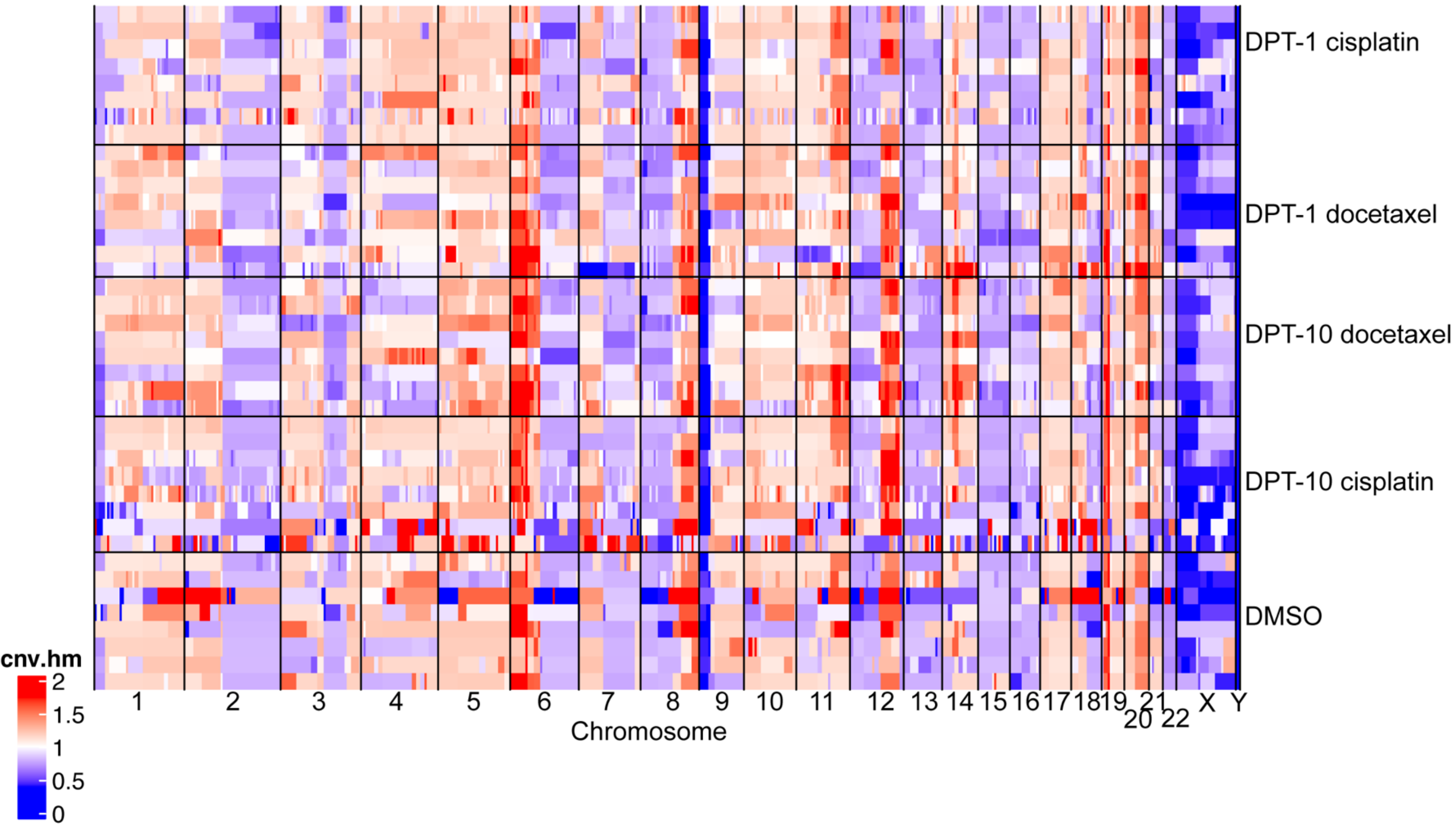

Figure S7: Segmented copy number ratios for MDA-MB-231 cells. X-axis represents chromosome location and Y depicts copy number ratio.

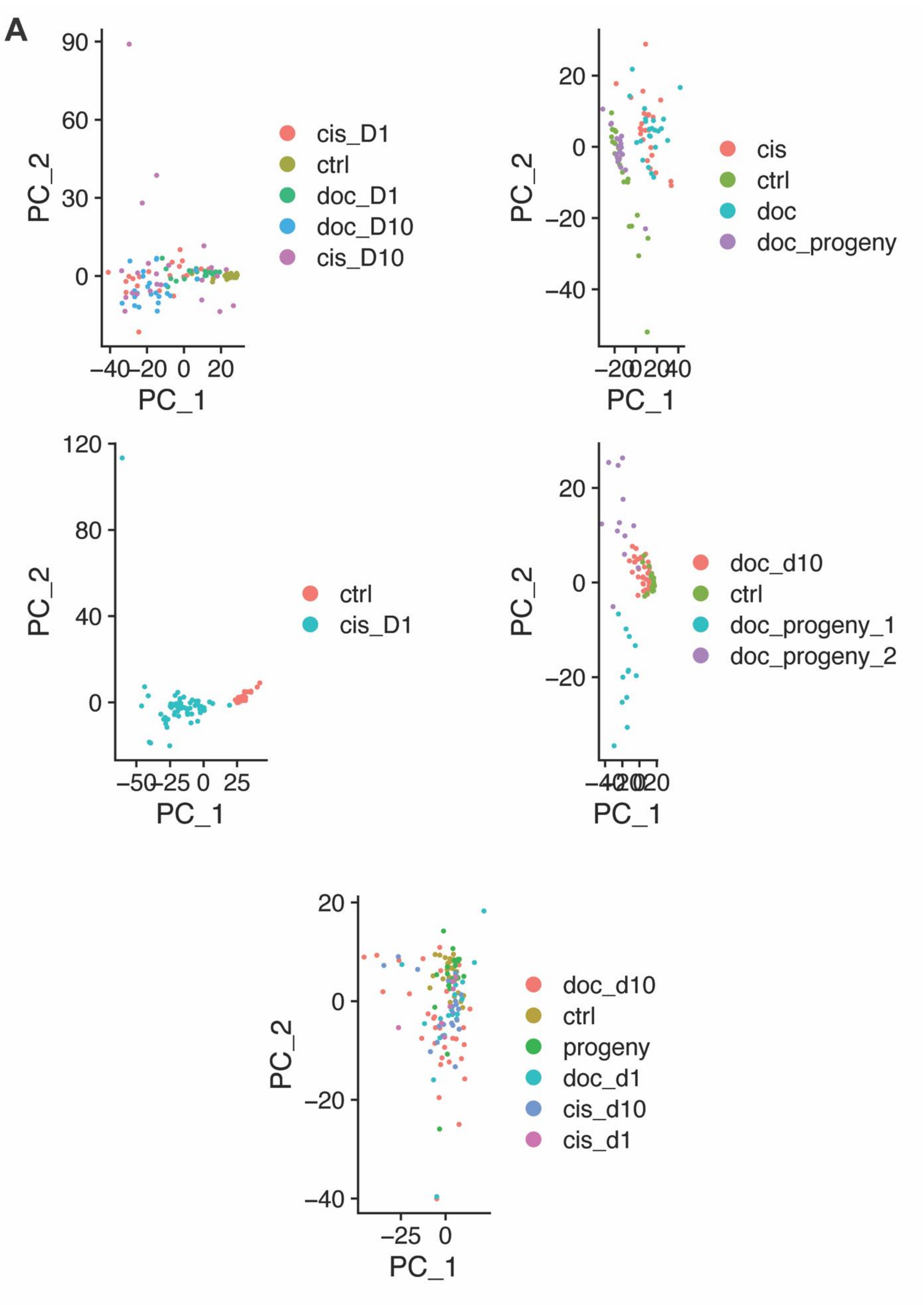

Figure S8: Principal components of different batches of RNA sequencing runs from PC3 conditions.

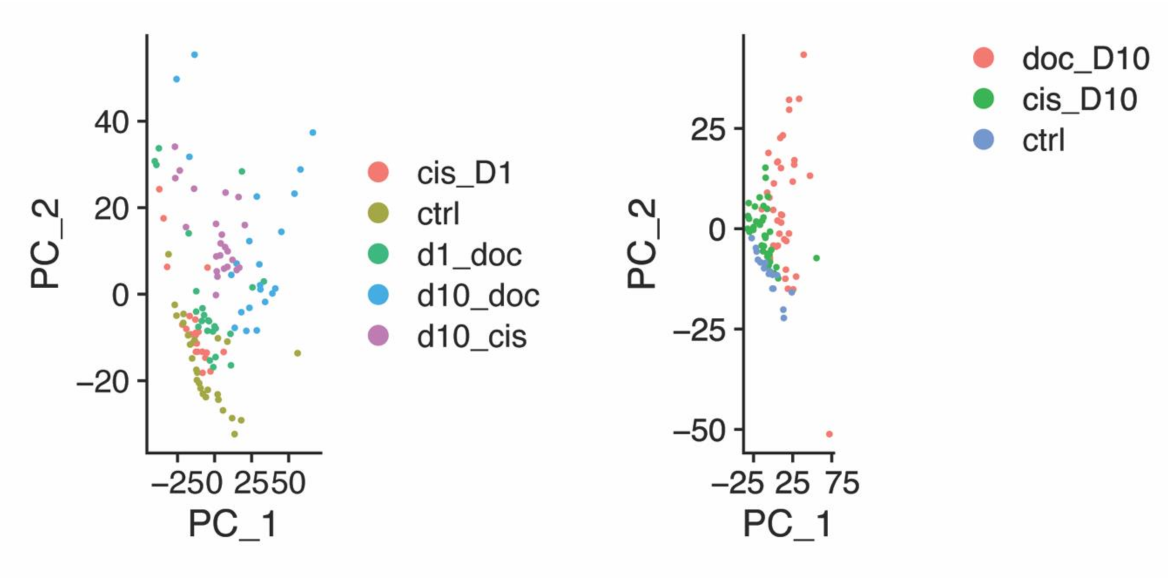

Figure S9: Principal components of different batches of RNA sequencing runs from PC3 conditions.

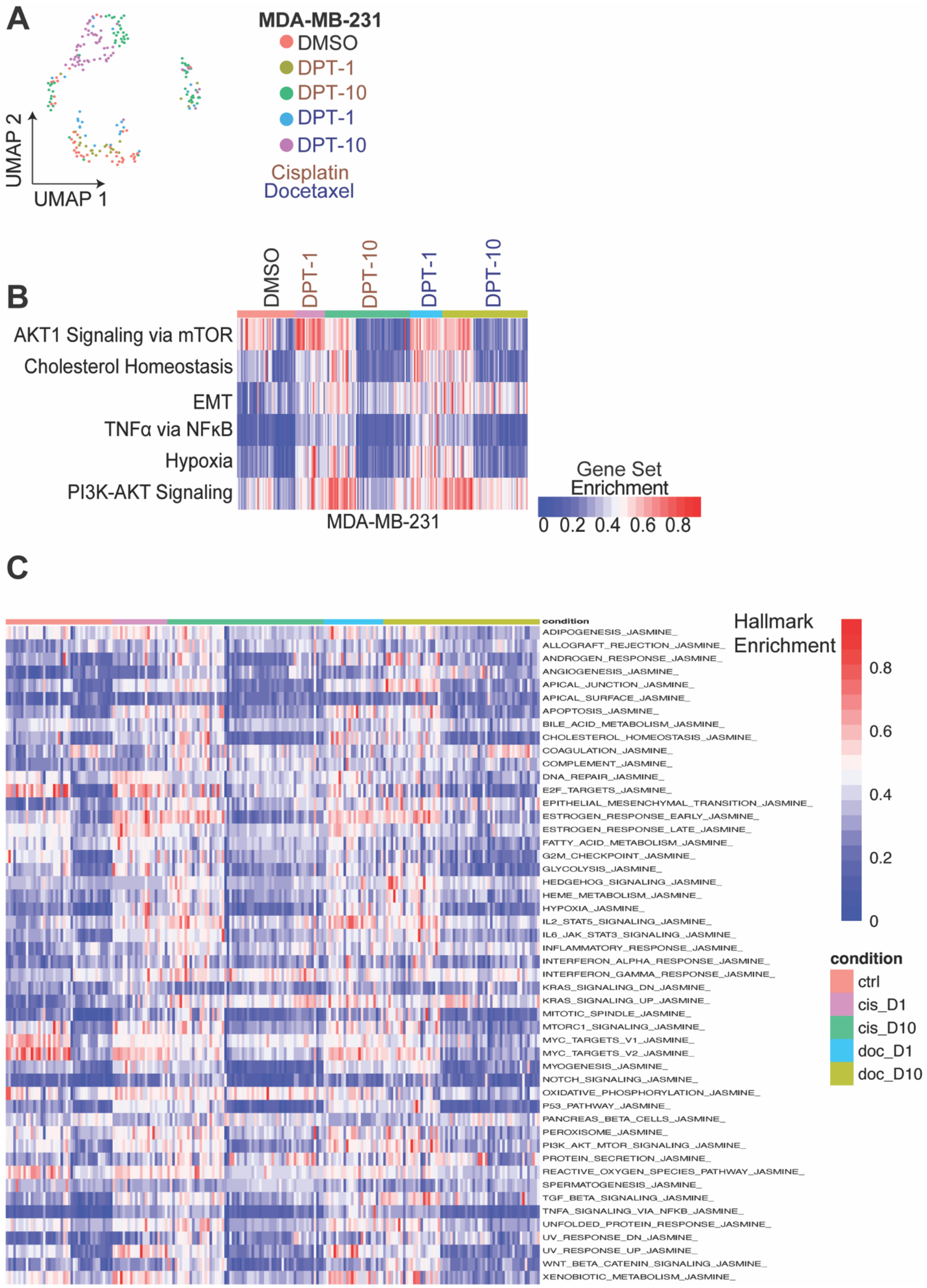

Figure S10: RNA sequencing of MDA-MB-231 cells. (A) UMAP visualization of MDA-MB-231 conditions. (B) Selected cell cycle hallmark pathway classifications as depicted in Figure 4H for PC3 cells (C) Full single cell hallmark pathway classifications.

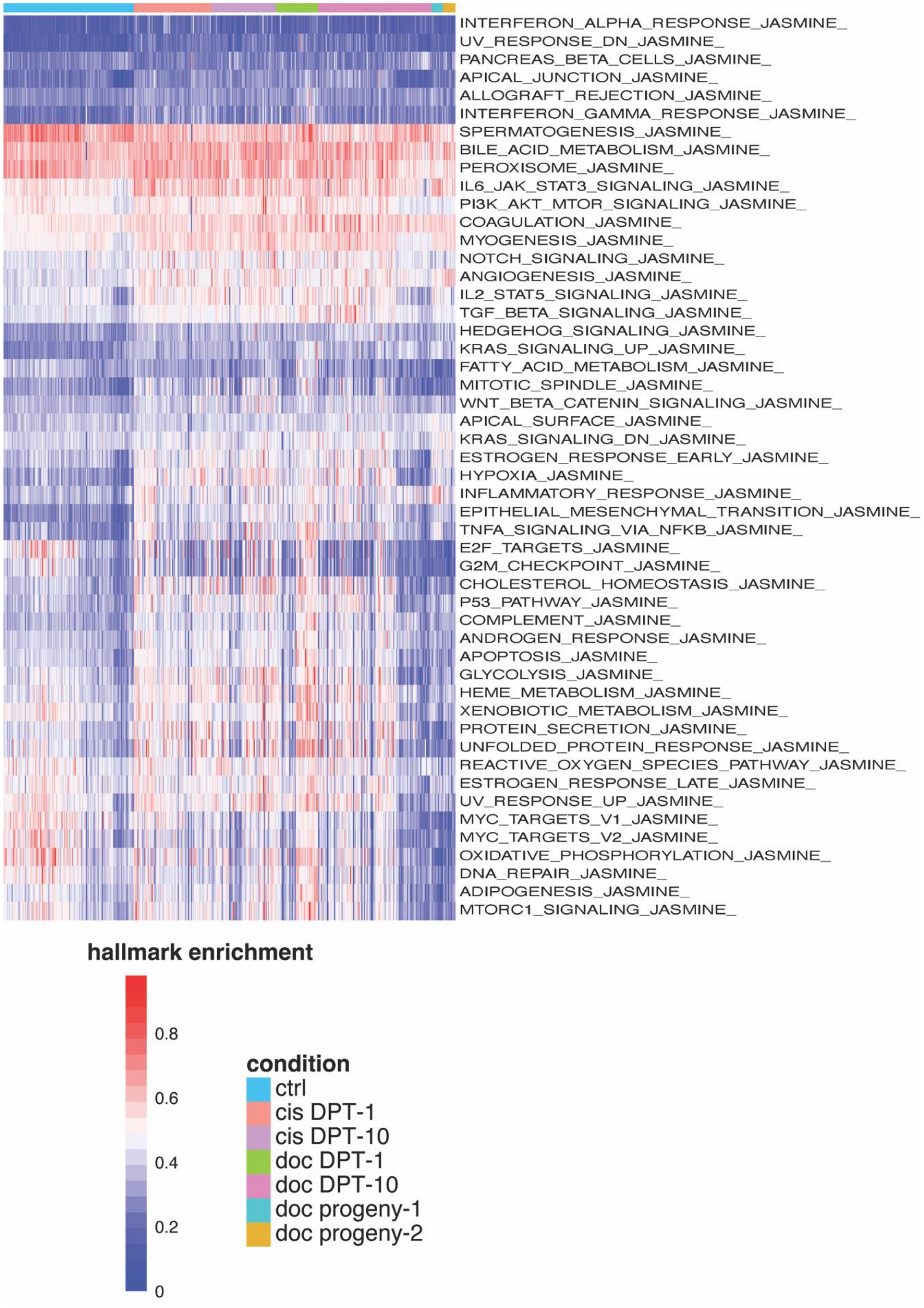

Figure S11: Full single cell hallmark pathway classifications for each condition for PC3 cells.

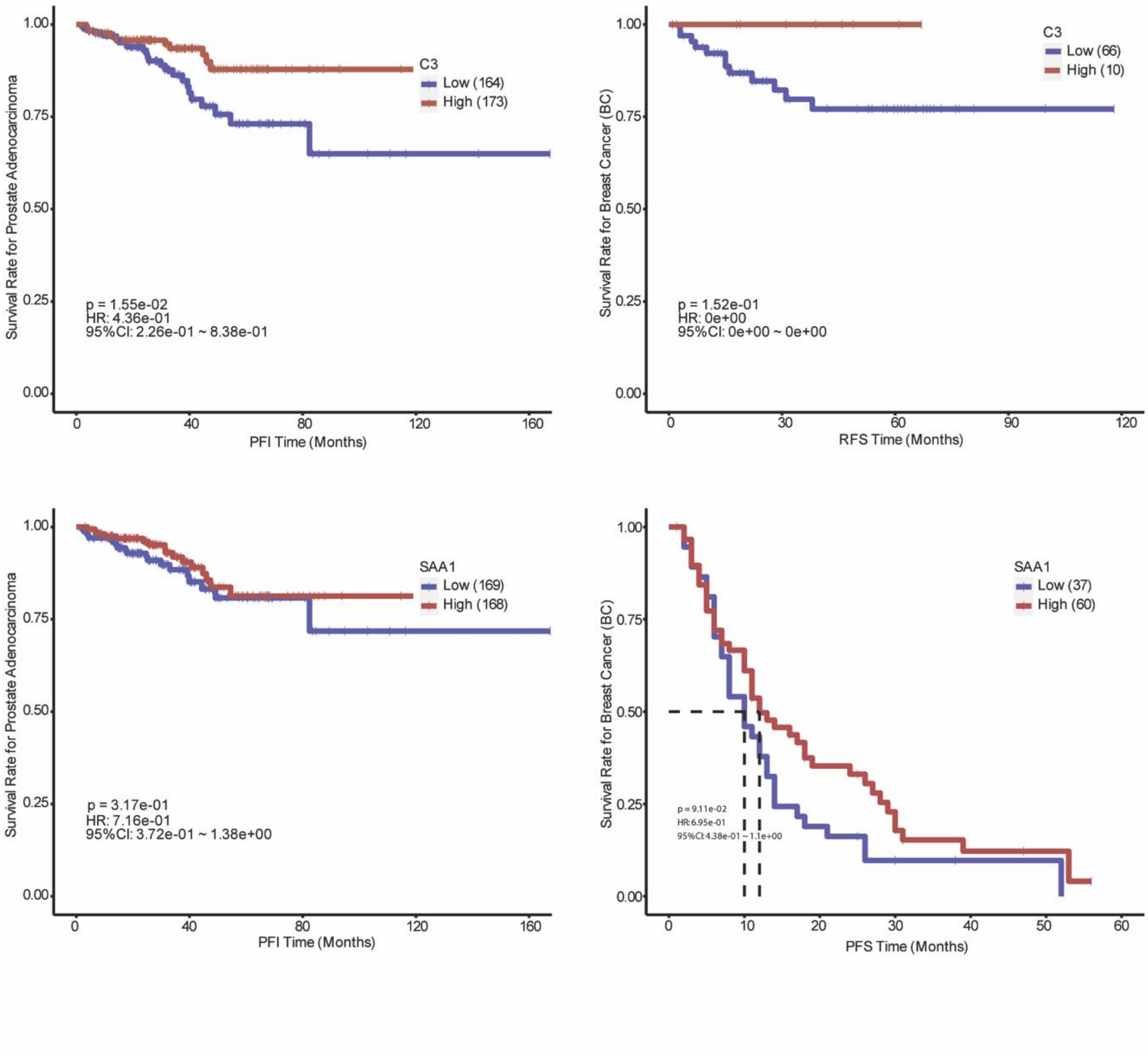

Figure S12: SAA1 and C3 are top DEGs in polyploid cancer cells (Figure 4D-E) but are correlated with better PFS in breast and prostate cancer patients.

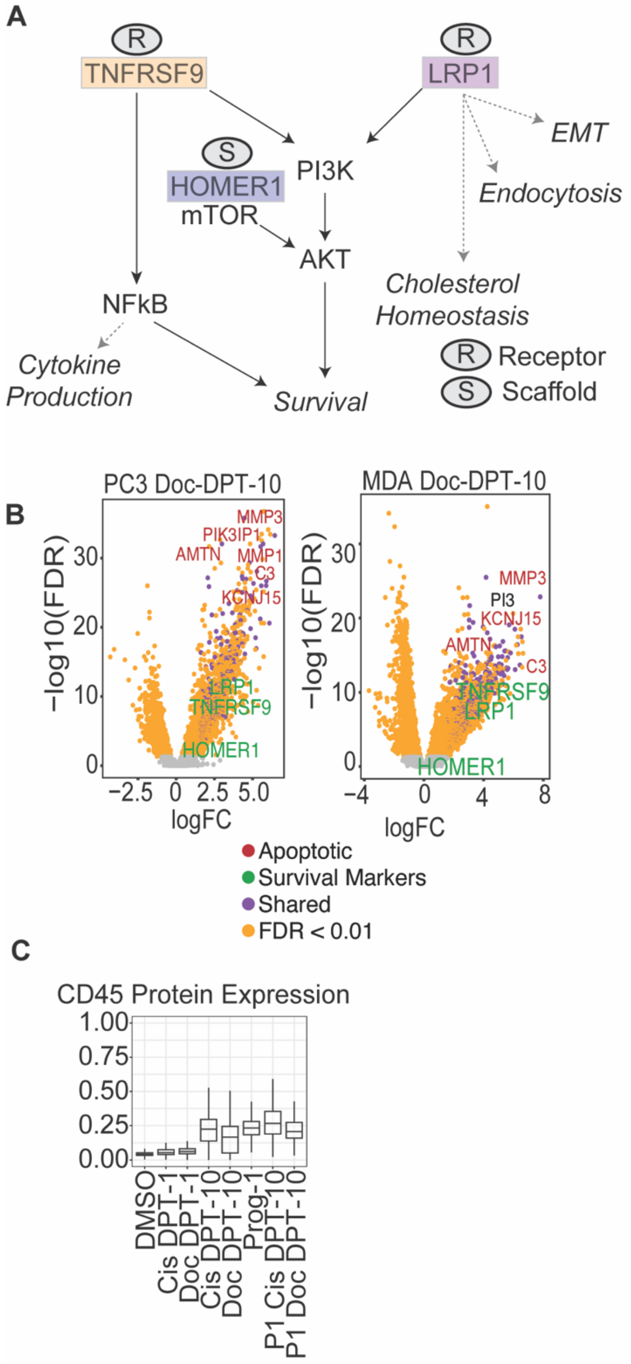

Figure S13: (A) Hypothesized involvement of TNFRSF9, HOMER1, and LRP1 in survival pathways for polyploid cancer cells. (B) Volcano plots for PC3 Docetaxel D10 and MDA-MB-231 Docetaxel DPT10 highlight top expressed genes and marker genes. (C) PC3 CD45 protein expression increases in chemotherapy DPT10 recovered cells and is retained in progeny.

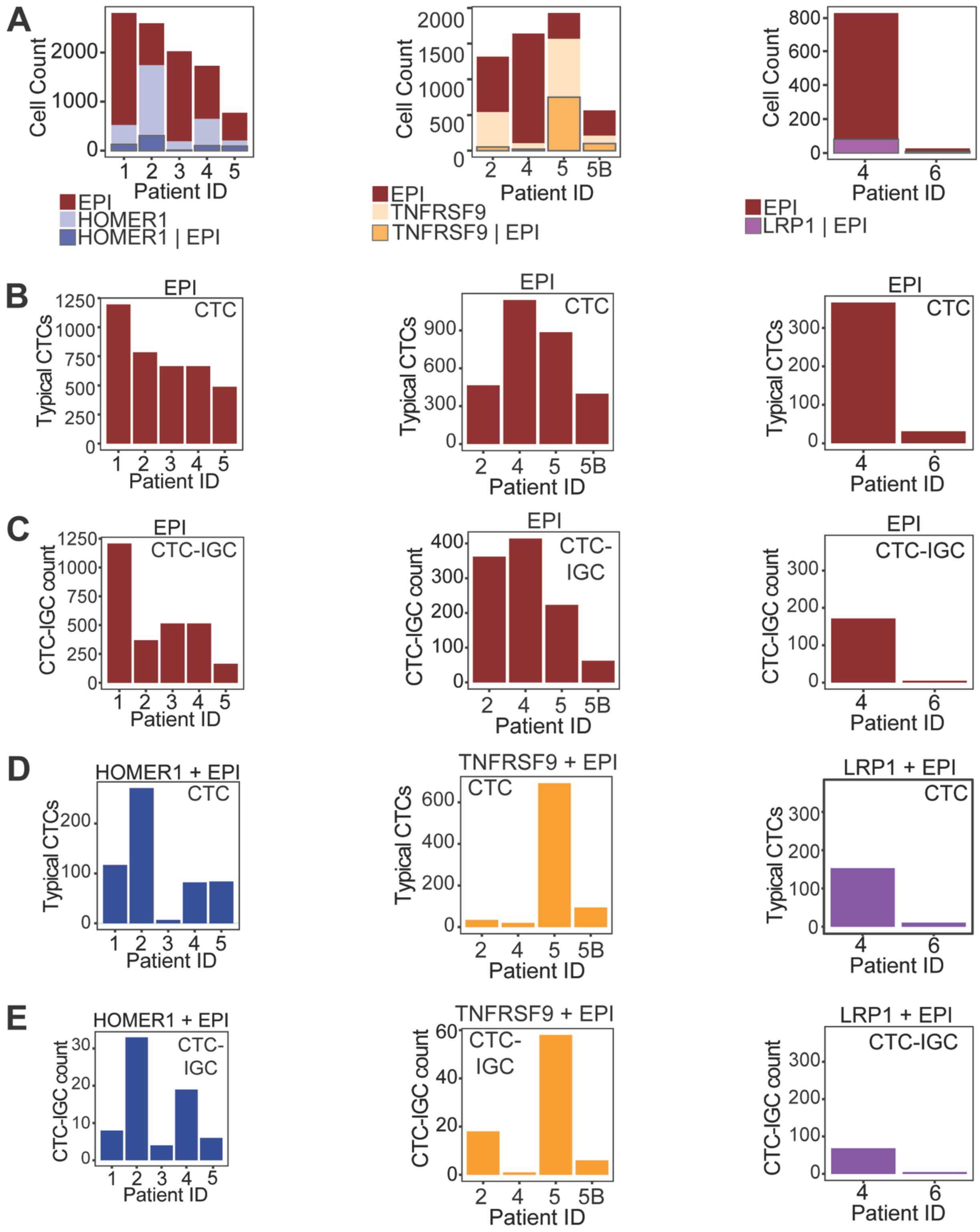

Figure S14: Biomarker staining in patient BM samples. (A) Total cell count for cells depicted in Figure 6B. rare cells with each channel positivity depicted. Cells were stained with survival markers HOMER1 (left), TNFRSF9 (middle), and LRP1 (right). Cells that have EPI positivity are tumor derived, while cells that are positive for the marker alone (middle bar in bar plot) cannot be conclusively labeled as a tumor derived cell. (B) Typical cell count count for regular sized cells that are only EPI positive and are not positive for the respective survival marker stained (above in A). (C) CTC-IGC count for polyploid cells that are only EPI positive and are not positive for the respective survival marker stained (above in B). (D) Typical CTC count for regular sized cells that are the survival marker and EPI positive. (E) CTC-IGC count for polyploid cells that are the survival marker and EPI positive.

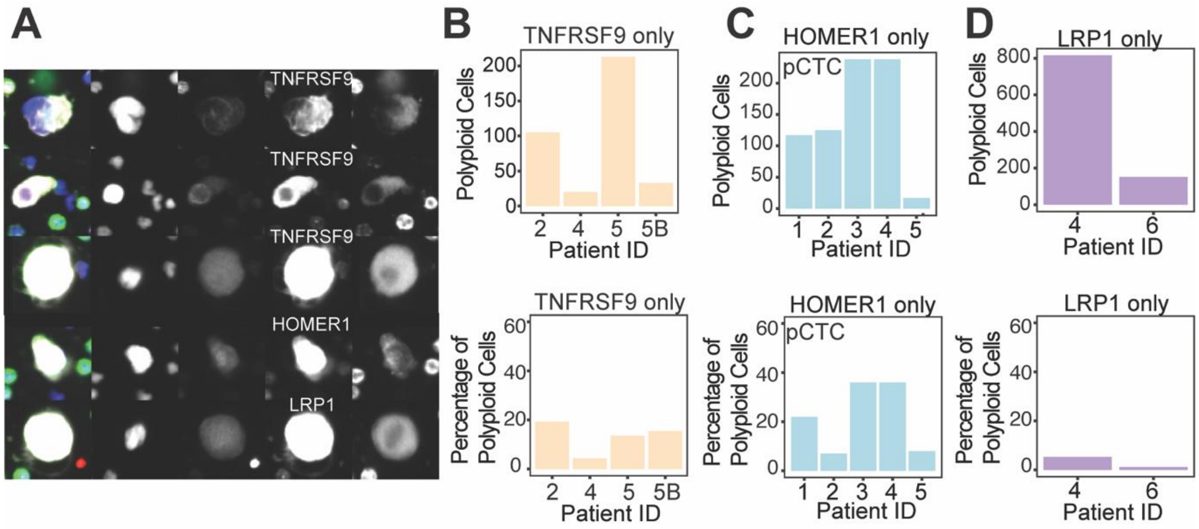

Figure S15: Biomarker staining in patient BM samples of polyploid cells that are positive for marker of interest but negative for EPI channel (not canonical CTCs). (A) Representative images for CTC-IGCs that are positive in survival marker channel (labelled in image) but negative for EPI channel. (B) Enumeration (top) and percentage (bottom) of polyploid cells that are positive for only the TNFRSF9 channel and not the EPI channel. (C) Enumeration (top) and percentage (bottom) of polyploid cells that are positive for only the HOMER1 channel and not the EPI channel. (D) Enumeration (top) and percentage (bottom) of polyploid cells that are positive for only the LRP1 channel and not the EPI channel.
